# Early Vertebroplasty for Severely Painful Acute Osteoporotic Compression Fractures: A Critical Review of the Literature

**DOI:** 10.1007/s00270-022-03348-z

**Published:** 2023-01-18

**Authors:** William Clark, Terrence Diamond

**Affiliations:** 1 Interventional Radiology, St George Private Hospital, Sydney, NSW Australia; 2 St George and Sutherland Clinical School, St George Hospital, University of NSW, Sydney, NSW Australia

**Keywords:** Vertebroplasty, Osteoporosis, Pain, Vertebral compression fracture

## Abstract

Vertebroplasty has emerged over the last 30 years as a common treatment for painful osteoporotic vertebral fractures. Patient selection and the time at which vertebroplasty is offered to the patient varies between centres and regions. Vertebroplasty has been studied in comparison to placebo intervention in five blinded trials. One such trial showed more benefit from vertebroplasty than placebo when the procedure was mostly performed within 3 weeks of fracture onset. Others showed no additional benefit from vertebroplasty compared to placebo when it was performed later in the natural history of the fracture. In this review, we examine data from blinded and open label randomised studies of vertebroplasty for evidence relating specifically to the use of early vertebroplasty for patients with severely painful acute osteoporotic fractures.

## Introduction

Vertebroplasty is the injection of polymethyl methacrylate (PMMA) into the trabecular bone of the vertebral body to stiffen the bone against compressive deformity. Since the first vertebroplasty was performed in 1984, it has evolved as a common treatment for painful osteoporotic vertebral compression fractures.

The vertebral body is the most common location for osteoporotic fractures. Many of these are noted as chronic vertebral deformities but some present as acute fractures. Patients are traditionally managed with simple analgesia and anti-osteoporotic therapies with the expectation that pain will gradually improve. Some elderly patients develop severe, intractable pain, which is difficult to control, and they may lose mobility, self-confidence or develop complications from opiate analgesia.

With the arrival of vertebroplasty as a treatment option, an approach developed which allowed 3 weeks or more of conservative therapy to allow time for the pain to settle. Vertebroplasty was considered for patients whose pain remained problematic and had not resolved. The success of this approach depends upon the presumption that vertebroplasty is equally effective regardless of fracture age, which is not supported by the evidence.

The first blinded study of vertebroplasty [1] recruited patients with fractures up to 12 months duration, explaining that “guidelines recommend vertebroplasty for fractures that have not responded to medical treatment. Typically, the duration of such fractures ranges from several weeks to several months or longer for fractures that have not healed.”

We have 20 years of experience using a different approach, offering early vertebroplasty to patients with uncontrolled severe pain. Our first vertebroplasty study in 2003 [2] stated that *we elected to perform the procedure as early as 1 to 2 weeks after fracture because many of the patients were unable to cope with the pain.* We later studied this clinical approach in the VAPOUR trial [3] which found vertebroplasty more effective than placebo when performed early, with most patients in the trial having fractures of 3 weeks or less at the time of procedure. In this review we will examine randomised trials which have compared vertebroplasty to either placebo or conservative care for evidence relating specifically to early vertebroplasty, performed within 3 weeks of fracture onset.

## Mechanics of Early Vertebroplasty

Fracture plasticity is central to the mechanics of early vertebroplasty. Severe pain from early fractures is often due to “dynamic mobility” of the vertebral body [4] which is compressed when the patient attempts to get out of bed, causing severe pain. These fractures may contain fluid filled clefts [5] which are evident as bright (liquid) or black (gas) cavities on fat supressed T2 weighted magnetic resonance images (MRI). The clefts represent the plane of maximal fracture mobility.

**Fig. 1:**
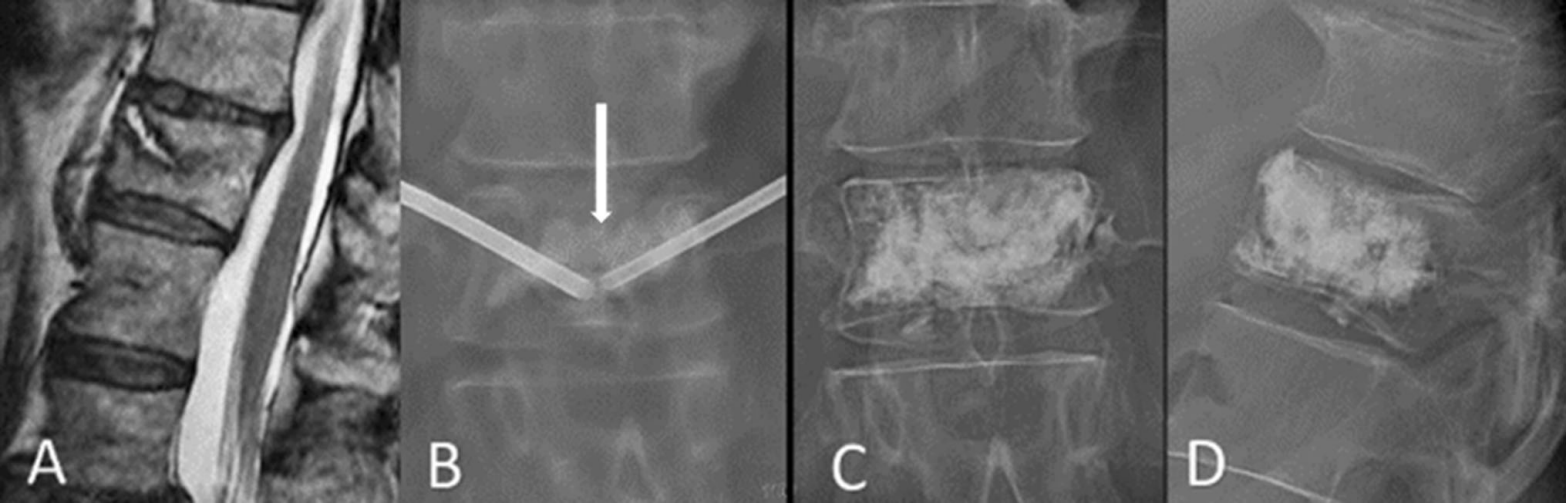
83-year-old female with 2 week history of severe back pain treated with vertebroplasty using “vertebral fill” technique. **A** Sagittal MRI shows oblique fracture cleft in T11. **B** Fluoroscopy image after injecting 3.5 cc of polymethyl methacrylate (PMMA) shows partial filling of the cleft (white arrow) but not the adjacent trabecular bone. **C** and **D**. AP and lateral images after vertebral fill injection of 8 cc PMMA. Cement is filling not just the fracture cleft but also supporting the trabecular bone above and below it to prevent ongoing fracturing. This is three times the mean PMMA volume injected in the earliest published blinded trials [1, 9], but similar to the mean volume used in the VAPOUR trial [3]

Vertebroplasty and more importantly the distribution of injected cement should be aimed at bracing the fractured vertebral body from top to bottom and side to side in order to resist these compressive forces on the vertebral body. This technical approach, called “vertebral fill” (Fig. 1), requires larger volume of polymethylmethacrylate than is feasible to inject into chronic fractures. There is minimal resistance to PMMA injection in early fractures which are characterised histologically by haematoma and osteonecrosis [6]. New bone and fracture callus develops progressively after 4 weeks preventing even distribution of PMMA and resulting in early leakage.

## Methodology

We review and summarise randomised trials comparing vertebroplasty to either placebo or conservative care, looking for data relating to early vertebroplasty, defined as vertebroplasty performed within 3 weeks of fracture onset. Outcomes of interest are pain, disability, and fracture morphology. Included trials were obtained from recently published systematic reviews [7, 8] which listed five blinded trials [1, 3, 9–11] comparing vertebroplasty to placebo and seven open trials comparing vertebroplasty to conservative care [12–19]. PubMed and clinicaltrials.gov were searched on August 23, 2022 for newer trials which had completed or been published since these reviews. We found one new trial had completed [20] but this studied chronic fractures and is unpublished.

### Blinded Trials of Vertebroplasty Versus Placebo Intervention

Five trials compared vertebroplasty to placebo intervention with participants blinded as to which treatment they received. The timing of vertebroplasty, inclusion criteria and mean PMMA volume injected per fracture varied (Table 1).

**Table 1:** Baseline data from five-blinded trials of vertebroplasty. Trials are arranged in increasing order of fracture age at time of vertebroplasty

Trial	No. (*n*)	Age (years)	BMD *T*-score	Fracture age (weeks)	Early vertebroplasty (%)	Inpatients (%)	PMMA (ml)
VAPOUR	120	80	− 4.3	2.6	79	57	7.5
VERTOS4	180	75	− 2.4	6	19	0	5.1
Hansen	46	70	− 2.4	NR	NR	0	NR
Buchbinder	78	74	NR	12	NR	0	2.8
Kallmes	131	73	NR	18	0	0	2.6

Early vertebroplasty = the percentage of vertebroplasty patients who received vertebroplasty within 3 weeks of fracture. Inpatients = the proportion of patients who were already hospitalised at the time of enrolment. PMMA volume = the amount of PMMA injected per treated fracture. Age, BMD, fracture age and PMMA volume are mean measurements. NR indicates that these data were not reported

*BMD* bone mineral density expressed as *T*-score

The first two blinded vertebroplasty trials [1, 9] were published together in 2009. Kallmes et al. [1] was the larger, enrolling 131 outpatients with fractures up to 12 months duration. Mean fracture duration was 18 weeks. Early vertebroplasty was excluded by clinical trial protocol which required a minimum of 3 weeks medical therapy before the patient could be enrolled. Mean PMMA volume was 2.6 cc suggestive of suboptimal fill technique.

Buchbinder et al. [9] enrolled 78 outpatients with back-pain for up to 12 months duration. Thirty-eight patients with mean fracture duration of 12 weeks underwent vertebroplasty. Mean PMMA volume was 2.8 cc suggestive of suboptimal fill technique. The Kallmes and Buchbinder trials found vertebroplasty no more effective than placebo in reducing pain.

VERTOS4 [10] recruited 180 outpatients who had been referred for spinal radiography and found to have a fracture. They completed a pain questionnaire assessing pain intensity and duration. Those with pain of 5 or more on a scale of 10 that had commenced within the previous 9 weeks were invited to enrol. They were then referred for MRI and physician consult to verify eligibility, adding an average of an extra 13 days, pushing the enrolment date and pain duration out to 11 weeks before intervention. Only 17 of the 90 patients who underwent vertebroplasty received it within 3.5 weeks of fracture (private communication). VERTOS4 found vertebroplasty and placebo equally effective in reducing pain.

Hansen et al. [11] recruited 46 participants with back pain persisting for up to 8 weeks through an outpatient orthopaedic clinic. No data on fracture duration is available. The primary endpoint summated back pain with forward flexion, assessed over multiple timepoints by repeated measure analysis and favoured vertebroplasty over placebo.

**Fig. 2:**
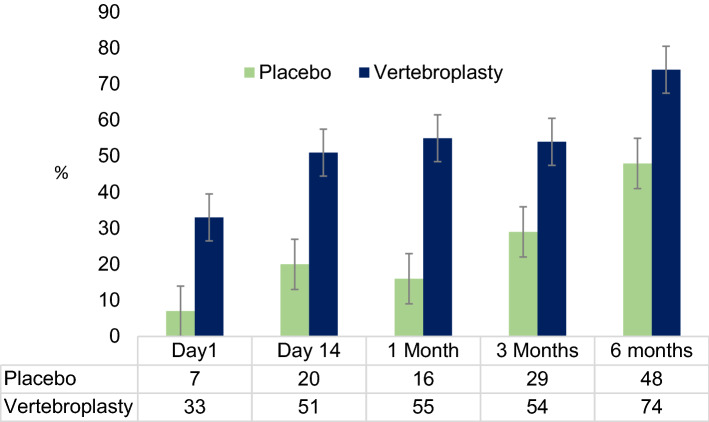
Primary outcome in VAPOUR subgroup of 93 patients with fractures ≤ 3 weeks duration at the time of intervention. Primary outcome is the proportion of patients with mild pain (NRS < 4 out of 10). Data table indicates the percentage of patients in each group achieving this primary outcome at each time point. All patients had severe pain (NRS ≥ 7 out of 10) at baseline. Vertical bars are 95% confidence intervals. This represents the only blinded outcome data for early vertebroplasty

The VAPOUR trial [3] enrolled 120 patients with pain of 7 or more out of 10 on numeric scale (NRS). Pain duration was restricted to 6 weeks, but most patients (79%) had early fractures of 3 weeks duration or less at time of vertebroplasty. Patients were older with more severe pain and more severe osteoporosis than other blinded trials (Table 1). VAPOUR was the only blinded trial to include hospital inpatients. PMMA volume per fracture was 7.5 cc reflecting the plasticity of early fractures allowing optimal fill technique. The primary outcome was the proportion of patients converting to a mild pain score (less than 4 on a scale of 10) and favoured vertebroplasty at all time points from 3 days to 6 months. The benefits were concentrated in patients receiving vertebroplasty within 3 weeks of fracture. There was no net benefit from vertebroplasty performed between 4 and 6 weeks. Improvement in disability scores also favoured vertebroplasty compared to placebo. Vertebral body height measures documented interval collapse in the control group and height restoration in the vertebroplasty group. Results in the subgroup with early fractures [21] represent the only blinded data for early vertebroplasty (Fig. 2).

**Fig. 3:**
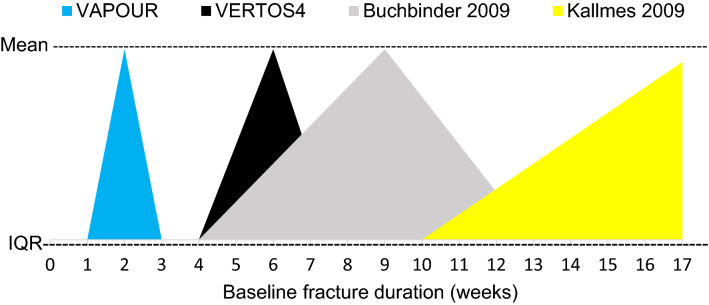
Fracture duration at the time of vertebroplasty in four blinded trials. The apex of each trial triangle is the median fracture duration at time of vertebroplasty and the base is the interquartile range (IQR) of fracture duration. The IQRs indicate that 75% of vertebroplasties occurred within 3 weeks of fracture in the VAPOUR trial compared to more than 75% occurring after 4 weeks in the other trials

Earlier intervention clearly differentiates VAPOUR from other blinded trials (Fig. 3). The interquartile range (IQR) of fracture duration is within 3 weeks of fracture onset in the VAPOUR trial and beyond 4-week in other blinded trials.

### Randomised Trials of Vertebroplasty Versus Conservative Therapy

Seven open label randomised trials compared vertebroplasty to conservative care (Table 2).

**Table 2:** Baseline data from seven open-label trials of vertebroplasty. Trials are arranged in increasing order of fracture age at time of vertebroplasty

Trial	No. (*n*)	Age (years)	BMD *T*-score	Fracture age (weeks)	Early vertebroplasty (%)	Inpatients (%)	PMMA (ml)
Yang	135	77	− 3.3	1.1	100	NR	4.5
Rousing	49	80	NR	1	80	most	NR
Klazen	202	75	− 3.0	6	NR	0	4.1
Leali	400	NR	NR	NR	NR	NR	NR
Voormolen	34	73	NR	12	0	0	3.2
Blasco	126	71	− 2.5	20	0	0	NR
Chen	96	65	NR	30	0	0	NR

Early vertebroplasty = the percentage of vertebroplasty patients who received vertebroplasty within 3 weeks of fracture. Inpatients = the proportion of patients who were already hospitalised at the time of enrolment. PMMA volume = the amount of PMMA injected per treated fracture. Age, BMD, fracture age and PMMA volume are mean measurements. NR indicates that these data were not published

*BMD* bone mineral density expressed as *T*-score

**Fig. 4:**
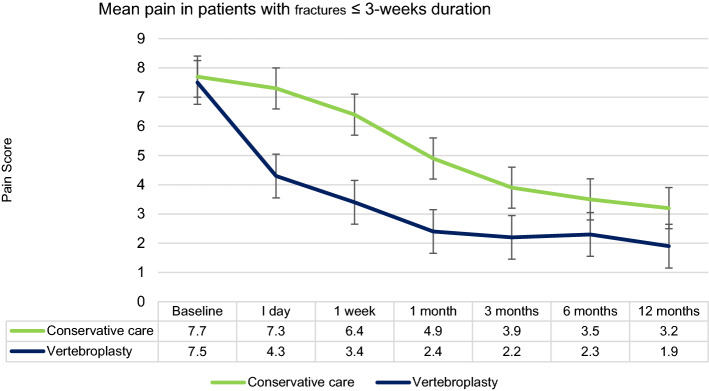
Mean visual analogue pain score (VAS) in the Yang 2016 trial of vertebroplasty which was the primary outcome. All patients had fracture duration of less than 3 weeks. The vertical bars represent 95% confidence intervals. Results favoured vertebroplasty over conservative care at all time points to 12 months. This trial represents open-label randomised trial data of early vertebroplasty within 3 weeks of fracture

Yang 2016 [12] randomised 135 patients aged more than 70 years with severe pain due to vertebral fracture less than 3-week duration. Vertebroplasty was performed an average 8 days after pain onset. Outcome data to 12 months follow up were available for 107 patients. Patients in the vertebroplasty group had greater reduction in pain and disability at all time points to 12 months (Fig. 4). Medical complications were more severe in the control group. Limitation to the trial was a single spine centre study.

Rousing 2009 [13] randomised 49 patients with painful fractures of less than 2 weeks duration in 80% and less than 8 weeks in the remainder. Problems with data collection resulted in a quarter of patients not having baseline data collected but they were still included in analysis. Baseline pain and quality of life measures differed between groups. Vertebroplasty reduced pain from 7.5 to 2.0 at 24 h but comparison measures were not recorded from the conservative group. Mean pain score at 3 months was the same in both groups. EQ5D was better in the vertebroplasty group but was also better at baseline. The vertebroplasty group was discharged from hospital 4 days earlier than the control group. Supplementary questionnaire [14], administered at 12 months, recorded a lower mean pain score (3.5) in the vertebroplasty group than in the control group (6.4), but this data are retrospective. Irregularities in data collection render the study difficult to interpret.

VERTOS2 [15] used similar enrolment pathway to VERTOS 4 except the limit of fracture duration was restricted to 6 weeks at time of radiograph rather than nine as in VERTOS4. 202 outpatients were randomised to vertebroplasty or conservative care. Mean fracture duration was 5.6 weeks (range 4 days–13 weeks) at the time of vertebroplasty. Data on the proportion of patients who underwent vertebroplasty within 3 weeks of fracture are not available. Baseline data were recorded at enrolment for the control group and nine days later, on the day of procedure, for the vertebroplasty group. There was significant baseline difference between groups in quality of life measures. The primary outcome of pain on a scale of ten was lower in the vertebroplasty group at all time-points to 12 months post procedure.

Leali 2016 [16] randomised four hundred women aged 55 to 82 with back pain for 6 weeks or less to single level vertebroplasty or usual care. The only data presented are baseline and 24 h pain and Oswestry Disability Index (ODI) scores which showed improvement in the vertebroplasty group but not the control group. No baseline demographics or other data is reported. The text states there was no difference between the two groups at other time points, but no data is provided.

Voormolen [17] excluded patients with fractures less than 6 weeks duration. Blasco 2012 and Chen 2014 [18, 19] were chronic fracture trials and can be discounted from early vertebroplasty analysis.

### Meta analyses

Meta-analysis of vertebroplasty is complicated by clinical differences between trials, most notably between VAPOUR and other blinded trials. Trials should not be pooled together if clinically heterogeneous.

The Cochrane protocol [22] for vertebroplasty meta-analysis states that “clinically heterogeneous studies will not be combined for analysis, but will be individually described”. The VAPOUR trial enrolled a different patient cohort, performed vertebroplasty earlier and used three times the volume of cement to the twin 2009 trials [1, 9] yet the Cochrane vertebroplasty report [8], written by authors of these 2009 trials, ignored the governing rules of the protocol and pooled VAPOUR with their own trials, to dilute the positive evidence supporting early vertebroplasty. This is one of multiple methodologic errors in the Cochrane vertebroplasty report, all of which act to nullify positive data for early vertebroplasty. The review found no evidence to support the efficacy of vertebroplasty regardless of fracture age which is clearly wrong. A more complete criticism of the motivation and methodology behind the Cochrane vertebroplasty report has been published [23].

Meta-analysis by Lou et al. [7] analysed VAPOUR as a separate sub-group, as recommended by Cochrane protocol, concluding that evidence supported the use of early vertebroplasty for patients with severe pain. Other meta-analyses [24–27] have generally supported the use of vertebroplasty particularly for fractures less than 6 weeks duration.

## Discussion

There are three randomised trials which have studied early vertebroplasty. The VAPOUR and Yang 2016 studies contain the most robust data. They enrolled similar patient cohorts who were aged over 70 years, had severe fracture pain and established osteoporosis. Vertebroplasty performed at a median fracture duration of 2-week in VAPOUR and 8-day in Yang provided superior pain and disability outcomes and less severe complications in both studies, compared to placebo or conservative treatment, respectively. Rousing 2009 is the third study to focus on early vertebroplasty but missing baseline data and other issues confound its analysis.

Blinded trials that reported equivalent outcomes from vertebroplasty and placebo performed vertebroplasty later than VAPOUR. Median fracture age at time of vertebroplasty was 2 weeks in VAPOUR, 6 weeks in VERTOS4, 9 weeks in Buchbinder and 18 weeks in Kallmes trials. Sub-group analysis within the VAPOUR trial found no benefit from vertebroplasty performed at 4 weeks or later, which aligns with findings in these other trials. Severely painful early fractures comprise blood and fluid filled osteonecrotic spaces and may be easily compressed in the upright position. Bone formation commences 2–4 weeks post fracture and progressive osteosclerosis occurs beyond 6 weeks [6]. It makes mechanical sense that effective prevention of fracture collapse is most likely to occur when vertebroplasty is performed while the fractured vertebral body comprises pools of blood and osteonecrotic spaces rather than new bone and fracture sclerosis. In this context, vertebral fill with PMMA can restore mechanical integrity to the vertebral body to prevent collapse.

VERTOS4 was another blinded trial with the next earliest fracture duration but it failed to demonstrate a benefit from vertebroplasty over placebo. Only a small minority of patients had early vertebroplasty. It is probable that a majority of patients in the study may have missed the window of opportunity for successful vertebroplasty. Patient selection parameters differed in additional ways. Most VAPOUR patients had been hospitalised with acute fractures and were recruited on clinical grounds whereas the VERTOS4 trial was an outpatient study only and recruited patients who had been referred for radiographs. The VERTOS4 patient cohort was younger and had better bone density measures.

The twin trials of 2009 by Kallmes and Buchbinder respectively paved the way for blinded research into vertebroplasty for which we should be grateful. They cannot, however, be construed to provide evidence for the efficacy of early vertebroplasty. The patient selection, timing of vertebroplasty and technique of vertebroplasty are entirely different to that of the VAPOUR study. Even were the vertebroplasties performed much earlier and on older patients with more severe pain and poorer bone density, the amount of PMMA used in these trials (2.6–2.8 cc) is insufficient to brace an early fracture of the vertebral body, as demonstrated in Fig. 1.

The placebo technique has been proposed as an explanation of different trial outcomes [10]. VAPOUR injected lidocaine under the skin before making an incision. Other trials injected additional lidocaine on the posterior surface of the pedicle, which is remote from the fracture. The hypothesis that this short acting local anaesthetic, with a duration of action of less than two hours, may have provided pain relief over weeks to months is unlikely. The fracture itself was not injected and there is no mechanism to explain an action more prolonged than the injected agent.

The significance of different PMMA volumes between trials needs clarification. The vertebral fill technique, described in the VAPOUR study, aims to brace the entire vertebral body not just the fracture cleft. Early osteoporotic fractures in elderly patients are soft and non-sclerotic and provide minimal resistance to needle introduction and PMMA injection. Mean PMMA volume injected in VAPOUR was 7.5 cc per bone and the frequency of minor extrusion was 37%. The Buchbinder et al. trial described its technical methodology thus: *Injection was stopped when substantial resistance was met or … if cement leaked into extraosseous structures or veins.* Mean PMMA volume in the Buchbinder trial was 2.8 cc which is 32% of the volume used in VAPOUR, yet the rate of minor extravasation (37%) was similar. These data are reflective of more sclerotic, older fractures preventing optimal vertebral fill technique. The Mean PMMA volume in VERTOS4 was 5.1 cc but at the cost of a remarkably high extrusion rate of 91% indicating fracture resistance.

Elderly patients hospitalised with acute osteoporotic vertebral fractures will often benefit from early vertebroplasty. VAPOUR is the only blinded trial to study vertebroplasty in this group. Hospitalisation normally occurs due to uncontrolled pain and loss of mobility causing an inability to cope at home. Pain is maximal when the patient attempts to climb out of bed and the fractured vertebra is compressed by the weight of the upper body. Hospitalised patients usually have a history of early fracture presentation, advanced age, co-morbidities and severe osteoporosis [28, 29]. They do poorly with conservative care and often suffer with increased morbidities and complications from opiate therapies. Vertebroplasty can interrupt this downward spiral and permit early rehabilitation and hospital discharge. The two trials which have studied duration of hospitalisation both found that vertebroplasty reduced hospital stay which may reduce health spending [3, 13].

VERTOS4 [10] raised a common concern about early intervention, that *in general, if vertebroplasty is performed too early then treatment will largely be for fractures that are destined to improve anyway by natural healing.* The VAPOUR trial showed that the natural history of elderly patients with severely painful early osteoporotic fractures was not positive. Patients in the placebo group had poor clinical outcomes to 6-months, with early intervention reducing pain, disability and the duration of hospitalisation in the vertebroplasty group. To avoid unnecessary intervention, early vertebroplasty should target patients with advanced age, severe symptoms, poor bone density and include hospitalised inpatients as in the VAPOUR trial. Calibrated vertebral height measurements demonstrated a 63% vertebral body height loss at 6-months in the placebo group as compared to 27% in the vertebroplasty group, outlining a distinct benefit in the natural history of fracture progression.

Early vertebroplasty is a procedure for patients whose advanced age and osteoporosis severely impairs fracture healing, resulting in fracture mobility, severe pain and loss of function. An early vertebroplasty programme requires MRI and intervention with minimal delay for patients with the most severe symptoms. This approach emulates the management of osteoporotic hip fractures. Access to MRI without delay needs to be prioritised for this patient group to allow the window of opportunity for early vertebroplasty and successful outcomes.

## Conclusion

Literature review supports the use of vertebroplasty in patients with severely painful fractures of 3 weeks duration or less. Data in the blinded VAPOUR trial and the open-label randomised trial by Yang and colleagues show that early vertebroplasty in selected patients is safe and effective. Future studies could further evaluate early vertebroplasty for patients with severely painful acute osteoporotic fractures, including hospitalised patients, to help guide patient selection and the optimal timing of vertebroplasty.
